# Targeting SphK1/2 by SKI-178 inhibits prostate cancer cell growth

**DOI:** 10.1038/s41419-023-06023-4

**Published:** 2023-08-21

**Authors:** Lu Jin, Jin Zhu, Linya Yao, Gang Shen, Bo-xin Xue, Wei Tao

**Affiliations:** 1 Department of Urology, the Second Affiliated Hospital of Soochow University, Suzhou, China; 2 Department of Urology, Kunshan Hospital of Traditional Chinese Medicine Affiliated to Yangzhou University, Kunshan, China; 3 Department of Urology, DUSHU Lake Hospital Affiliated to Soochow University, Suzhou, China

**Keywords:** Targeted therapies, Prostate cancer

## Abstract

Sphingosine kinases (SphK), including SphK1 and SphK2, are important enzymes promoting progression of prostate cancer. SKI-178 is a novel and highly potent SphK1/2 dual inhibitor. We here tested the potential anti-prostate cancer cell activity of SKI-178. Bioinformatics analyses and results from local tissues demonstrated that that both SphK1 and SphK2 are upregulated in human prostate cancer tissues. Ectopic overexpression of SphK1 and SphK2, by lentiviral constructs, promoted primary prostate cancer cell proliferation and migration. In primary human prostate cancer cells and immortalized cell lines, SKI-178 potently inhibited cell viability, proliferation, cell cycle progression and cell migration, causing robust cell death and apoptosis. SKI-178 impaired mitochondrial functions, causing mitochondrial depolarization, reactive oxygen species production and ATP depletion.SKI-178 potently inhibited SphK activity and induced ceramide production, without affecting SphK1/2 expression in prostate cancer cells. Further, SKI-178 inhibited Akt-mTOR activation and induced JNK activation in prostate cancer cells. Contrarily, a constitutively-active Akt1 construct or the pharmacological JNK inhibitors attenuated SKI-178-induced cytotoxicity in prostate cancer cells. In vivo, daily intraperitoneal injection of a single dose of SKI-178 potently inhibited PC-3 xenograft growth in nude mice. SphK inhibition, ceramide production, ATP depletion and lipid peroxidation as well as Akt-mTOR inactivation and JNK activation were detected in PC-3 xenograft tissues with SKI-178 administration. Together, targeting SphK1/2 by SKI-178 potently inhibited prostate cancer cell growth in vitro and in vivo.

## Introduction

Prostate cancer is the second most common malignancy among males [1, 2]. It is estimated that over 1.2 million new cases of prostate cancer and over 350,000 related death will occur each year [1, 2]. Over the past decade, significant progresses have been achieved in the clinical treatments for prostate cancer, including surgical resection, chemotherapy, immunotherapy and targeted therapies [3–5]. For the patients with metastatic, recurrent and other advanced prostate cancer, the prognosis and overall survival are however very poor [3–5]. To further explore the intercellular communications and signaling mechanisms for prostate cancer progression is essential for the development of molecularly-targeted and personalized therapies for advanced prostate cancer [3–5].

Sphingosine kinases (SphK), including SphK1 and SphK2, are key enzymes phosphorylating sphingosine to sphingosine 1-phosphate (S1P) [6–9]. Increased expression and/or activation of SphK1/2 often correlate with the poor prognosis in various human cancers [6–9]. In addition, SphK-S1P elevation could promote cancer cell survival, proliferation, angiogenesis, migration, invasion, and metastasis [6–9]. Conversely, pharmacological inhibition, genetic silencing or loss-of-function mutation of SphK1/2 could induce sphingosine and ceramide accumulation, causing growth arrest and apoptosis in different cancer cells [6–9].

Overexpression of SphK1 in prostate cancer is correlated with cancer grade and poor overall survival [10]. Lee et al., showed that lipopolysaccharide (LPS) promoted prostate cancer cell invasion and metastasis by activating SphK1 cascade [10]. LPS induced SphK1 S225 phosphorylation in a Toll-like receptor 4 (TLR4)-dependent manner, causing the translocation of SphK1 to plasma membrane, leading to S1P production and ERK1/2 activation, which were required for prostate cancer cell invasion and metastasis [10]. Gestaut et al., showed that SphK2 inhibition by a novel inhibitor, ABC294640, decreased viability and proliferation of androgen resistant prostate cancer cells [11]. These results implied that SphK1/2 are both important therapeutic targets of prostate cancer.

Recent studies have developed a novel SphK1 and SphK2 dual inhibitor SKI-178, competing for sphingosine binding site in SphK1/2 [12–15]. SKI-178 effectively reduced S1P formation while inducing ceramide accumulation in cancer cells [12–15]. As compared to other SphK inhibitors, it was significantly more cytotoxic against cancer cells [12–15]. Its potential effect in prostate cancer is tested here.

## Materials and methods

Chemical and reagents. Culture medium, N-Acetyl-L-cysteine (NAC) and ATP, fetal bovine serum (FBS) and antibiotics as well as the caspase-3 inhibitor z-DEVD-fmk and the pan caspase inhibitor z-VAD-fmk were from Invitrogen (Suzhou, China). Poly-d-Lysine was from BD Biosciences (Shanghai, China). Cell counting kit -8 (CCK-8) was provided by Dojindo Co. (Kumamoto, Japan). EdU (5-Ethynyl-2’-deoxyuridine) and TUNEL (Terminal deoxynucleotidyl transferase dUTP nick end labeling) and JC-1 (5,5’,6,6’-Tetrachloro-1,1’,3,3’-tetraethyl-imidacarbocyanine),were from Thermo-Fisher Invitrogen (Shanghai, China). Antibodies for SphK1 (#12071), SphK2 (#32346), cleaved caspase antibody sampler kit (#9929) and β-Tubulin (#2146) were purchased from Cell Signaling Technologies (Beverly, MA). SP600125 and JNK inhibitor II (JNKi-II) were obtained from Selleck (Shanghai, China).

### Cells and tissues

The established PC-3/LNCaP prostate cancer cell lines and the non-transformed RWPE1 prostate epithelial cell line were reported previously [16]. The primary prostate cancer cells (pCan-1 and pCan-2, derived from two written-informed consent patients) and the prostate epithelial cells were obtained and cultivated as described [16]. The primary pCan-1 and pCan-2 cells were derived from CRPC patients and were *PTEN*-null, *PI3KCA*-positive and *androgen receptor*-positive. Short-tandem repeat (STR) profiling, population doubling time, and cell morphology were always checked for the cells. The human prostate cancer tissues and matched adjacent normal prostate tissues of ten (10) written-informed consent castration-resistant prostate cancer (CRPC) patients were provided by Dr. Mi [17]. All protocols testing human cells/cells were approved by the Ethic Committee of Soochow University, in accordance to the principles expressed in the Declaration of Helsinki.

### Caspases assay

Cells were seeded in 96-well plates and treated with SKI-178. Afterwards, 40 μL of caspases-3-Glo substrate or the caspase-9-Glo substrate (Promega, Madison, WI) was added into each well for 1 h. A FlexStation 3 microplate reader (Molecular Devices, Sunnyvale, CA) was utilized to quantify Glo luminescence.

### Gene and protein detection

Western blotting and The quantitative reverse transcription PCR (qRT-PCR) protocols were described early [16]. Figure S1 included the uncropped blotting images.

### shRNA

The GV369 lentiviral vectors encoding SphK1 shRNA and SphK2 shRNA were synthesized by Shanghai Genechem Co (Shanghai, China). The construct, together with the lentivirus Helper plasmids, was co-transfected to HEK-293T cells (Genechem). The generated lentivirus were added to prostate cancer cells (cultured in polybrene medium) for 24 h. Cells were then cultured in puromycin (5 μg/mL)-containing complete medium for another 48 h, and stable cells established. SphK1/2 expression in stable cells was tested by Western blotting and qRT-PCR assays. The scramble control shRNA lentivirus (Genechem, Shanghai, China) was added to the control prostate cancer cells.

### Constitutively-active mutant Akt1

The lentivirus-packed constitutively-active Akt1 (caAkt1, S473D) was reported in our previous study [16]. It was transduced to cultured prostate cancer cells for 48 h. Stable cells were formed by puromycin selection. Expression of caAkt1 was verified by Western blotting assays.

### SphK1/2 overexpression

The GV369 lentiviral SphK1-expressing construct and the GV369 lentiviral SphK2-expressing construct were both provided by Genechem (Shanghai, China). Each construct was transfected to HEK-293 cells along with lentiviral packaging constructs (Genechem), thereby generating lentivirus. The virus was then filtered and enriched, and was added (at MOI = 15) to cultured prostate cancer cells (in polybrene medium). Stable cells were then established after selection using puromycin. SphK1/2 overexpression was verified by the Western blotting assays. Control cells were infected with vector control lentivirus.

### Transwell assays

Cells in no serum basic medium were added in the upper surface of the chamber (at 1 × 10^4^ cells per chamber) and were allowed to migrate for 16 h. Cells on the lower surface were then fixed, stained and were photographed by an microscope.

### Other assays

Cell viability by CCK-8 assay, colony formation, the nuclear TUNEL staining and the nuclear EdU staining assays were reported in our previous study [16]. CellROX staining of reactive oxygen species (ROS) contents, single strand DNA (ssDNA) ELISA, JC-1 staining assay of mitochondrial depolarization, and lipid peroxidation measurement by thiobarbituric acid reactive substances (TBAR) activity assay were described in detail in other studies [18, 19]. Measuring the ATP contents in cellular/tissue lysates as well as testing the mitochondrial complex I activity in cellular lysates were described early [20, 21].

### Xenograft studies

The protocols of the animal studies were in according to the national and international regulations, with approval from the Institutional Animal Care and Use Committee (IACUC) and Ethic Committee of Soochow University. The nude mice (5–6 weeks old, all male, 18.5–19.5 g) were obtained from the Animal Center of Soochow University. Mice were housed under standard procedures and were injected subcutaneously (s.c.) with PC-3/pCan1 cells (at six million cells/mice) in Matrigel-serum medium. Tumor volumes were measured by the modified ellipsoid formula: (π/6) × *AB*^2^, with *A* the longest and *B* the shortest perpendicular axis of a tumor mass [22]. Tissue slides were tested via immunohistochemistry (IHC) staining and immuno-fluorescence assays using the described protocols [23].

Statistical analyses. Experimental data were expressed as mean ± standard deviation (SD). The statistical significance of differences between the means of two treatment groups was evaluated by unpaired two sided Student’s *t*-test (Excel 2007), and overall significance of multiple groups was evaluated by ANOVA followed by Tukey’s multiple comparison test of variance (SPSS 23.0, SPSS, Chicago, CA). All statistical tests were two-sided and the level of significance was set at *P* < 0.05.

## Results

### SphK1 and SphK2 are upregulated in human prostate cancer tissues

**Fig. 1:**
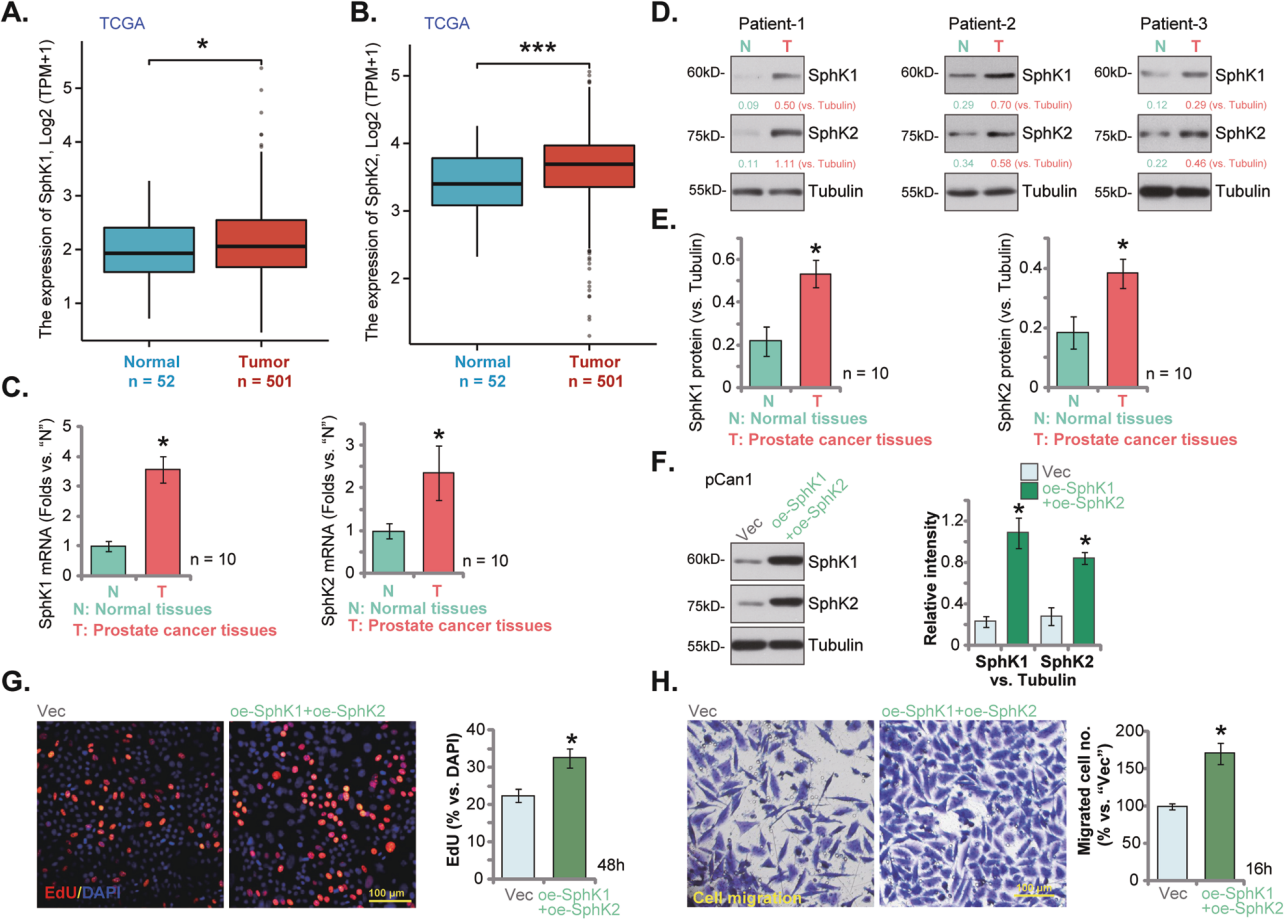
SphK1 and SphK2 are upregulated in human prostate cancer tissues. TCGA database shows the number of *SphK1* transcripts (**A**) and the number of *SphK2* transcript (**B**) in 501 prostate cancer tissue samples (“Tumor”) and 52 para-cancerous normal prostate tissue samples (“Normal”). Expression of *SphK1 and SphK2* mRNA (**C**) and listed proteins (**D**, **E**) in human prostate cancer tissues (“T”, derived from 10 different CRPC patients, *n* = 10) or cancer-surrounding normal prostate tissues (“N”) were shown. The primary human prostate cancer cells pCan1, with the lentiviral SphK1-overexpressing construct plus the lentiviral SphK2-overexpressing construct (“oe-SphK1+oe-SphK2”) or the empty vector (“Vec”), were established and expression of listed proteins was tested (**F**); Cells were further cultivated for indicated time periods, cell proliferation (by measuring nuclear EdU staining, **G**) and cell migration (“Transwell” assays, **H**) were measured. Data were expressed as the mean ± standard deviation (SD). “TPM” stands for transcripts per million. ***P < 0.001 versus “Normal” tissues. *P < 0.05 versus “Normal” or “N” tissues (**C**–**E**). *P < 0.05 versus “Vec” cells (**F**–**H**). Scale Bar = 100 μm.

The Cancer Genome Atlas (TCGA) database was first consulted to examine *SphK1/2* expression in human prostate cancer. A total of 553 tissues samples were collected, including 501 prostate cancer tissue samples and 52 para-cancerous normal prostate tissue samples. As shown in Fig. 1A, the number of *SphK1* transcripts in the prostate cancer tissues (“Tumor”) was significantly higher than that in the normal prostate tissues (“Normal”). Moreover, upregulation of *SphK2* transcripts was detected in the prostate cancer tissues from the TCGA database (Fig. 1B).

The expression of SphK1/2 in local prostate cancer tissues was examined as well. As demonstrated, the mRNA expression of both *SphK1* and *SphK2* was significantly elevated in human prostate cancer tissues (“T”) from 10 different CRPC patients (tissues were provided by Dr. Mi [17]) (Fig. 1C). The protein expression of both SphK1 and SphK2 was upregulated in prostate cancer tissues of three representative patients (“Patient-1/-2/-3”) (Fig. 1D). Their expression was however relatively low in cancer-surrounding normal prostate tissues (“N”, Fig. 1D). When combining all 10 sets of tissue blotting data, we found that SphK1 and SphK2 protein upregulation was significant in the local prostate cancer tissues (Fig. 1E). These results show that both SphK1 and SphK2 are upregulated in human prostate cancer tissues.

To support the role of SphK1 and SphK2 in the growth of prostate cancer cells. The lentiviral SphK1-overexpressing construct and the lentiviral SphK2-overexpressing construct were co-transduced to pCan1 primary prostate cancer cells, and stable cells were established after selection: “oe-SphK1+oe-SphK2” pCan1 cells. As compared to control cells with empty vector (“Vec”), the protein expression of both SphK1 and SphK2 was significantly elevated in the oe-SphK1+oe-SphK2 pCan1 cells (Fig. 1F). In SphK1/2-overexpressed pCan1 cells, cell proliferation (nuclear EdU incorporation, Fig. 1G) and cell migration (Fig. 1H) were robustly augmented. These results supported the important role of SphK1/2 overexpression in prostate cancer cell growth.

### SKI-178 exerts significant anti-cancer activity in cultured prostate cancer cells

**Fig. 2:**
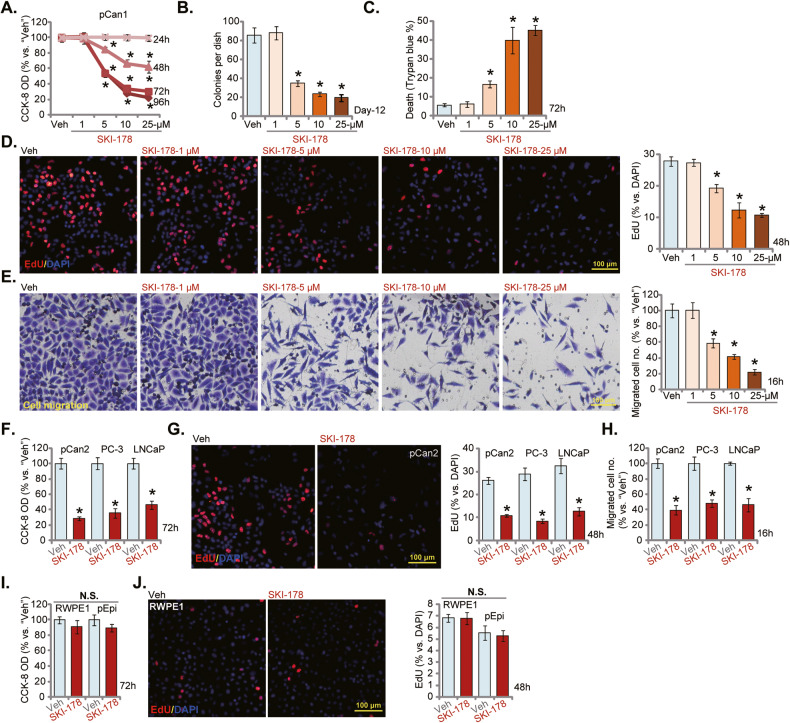
SKI-178 exerts significant anti-cancer activity in cultured prostate cancer cells. The primary human prostate cancer cells (“pCan1” and “pCan2”) (**A**–**H**), the established cell lines (PC-3 and LNCaP) (**F**–**H**), the primary human prostate epithelial cells (“pEpi”) (**I** and **J**) or the established RWPE-1 epithelial cells (**I**, **J**) were cultivated in complete medium and treated with SKI-178 (10 μM or at designated concentrations for **A–E**) for indicated time periods; Cell viability (CCK-8 assay, **A**, **F** and **I**), colony formation (**B**), cell death (Trypan blue staining assays, **C**) proliferation (nuclear EdU staining assays, **D**, **G**, and **J**), and cell migration (“Transwell” assays, **E** and **H**) were tested by the assays mentioned in the text. “Veh” stands for vehicle control. Values represented the mean ± SD (*n* = 5). *P < 0.05 versus “Veh” group. Experiments in this figure were repeated five times, with similar results achieved. Scale Bar = 100 μm.

The primary human prostate cancer cells, pCan1, were cultivated in completed medium and treated with SKI-178 at gradually increased concentrations (from 1-25 μM). Cells were then cultured for different periods, and cell viability examined by CCK-8 assays. As shown, SKI-178 potently inhibited pCan1 cell viability (CCK-8 OD) in a concentration-dependent manner (Fig. 2A). SKI-178 was significant at 5-25 μM in decreasing pCan1 cell viability (Fig. 2A). It was ineffective at 1 μM (Fig. 2A). Moreover, SKI-178 displayed a time-dependent response and would require at least 48 h to exert a significant anti-survival activity against pCan1 cells (Fig. 2A). Its effect lasted for at least 96 h (Fig. 2A). In Fig. 2B, the colony formation assay results showed that SKI-178 (5-25 μM) significantly decreased the number of viable pCan1 cell colonies. To further support the cytotoxic effect, we showed that the number of “dead” pCan1 cells, with Trypan blue-positive staining, was significantly increased after SKI-178 (5-25 μM) treatment (Fig. 2C).

The EdU staining assays were employed to test cell proliferation. SKI-178 efficiently decreased the EdU-positive nuclei ratio in pCan1 cells (Fig. 2D), supporting its anti-proliferative activity. Further experimental results showed that SKI-178 dose-dependently inhibited pCan1 cell in vitro migration (Fig. 2E) assays, respectively. Since 10 μM of SKI-178 induced robust anti-prostate cancer activity (Fig. 2A-E), this concentration was selected for the following studies.

The potential effect of the SphK1/2 dual inhibitor on other prostate cancer cells was examined as well. The primary human prostate cancer cells-derived from another patient, pCan-2, as well as the established cell lines, PC-3 and LNCaP, were treated with SKI-178 (10 μM) and cultured for applied time periods. As shown SKI-178 potently decreased the cell viability (CCK-8 OD) in the prostate cancer cells (Fig. 2F). Results from EdU staining (Fig. 2G) and Transwell (Fig. 2H) assays demonstrated that SKI-178 significantly inhibited proliferation and migration of the primary and established prostate cancer cells. These results showed that SKI-178 exerted potently anti-cancer activity in prostate cancer cells by inhibiting cell viability, proliferation and cell migration.

Next experiments were performed to examine the potential effect of SKI-178 in non-cancerous prostate epithelial cells. The primary human prostate epithelial cells (“pEpi”) and established RWPE-1 cells [24, 25] were treated with SKI-178. Figure 2I showed that SKI-178 (10 μM, 72 h) failed to significantly inhibit cell viability of RWPE-1 cells and primary prostate epithelial cells. Moreover, the SphK1/2 dual inhibitor did not inhibit prostate epithelial cell proliferation, as the EdU-positive nuclei ratio was not significantly changed (Fig. 2J).

### SKI-178 induces cell cycle arrest and apoptosis in prostate cancer cells

**Fig. 3:**
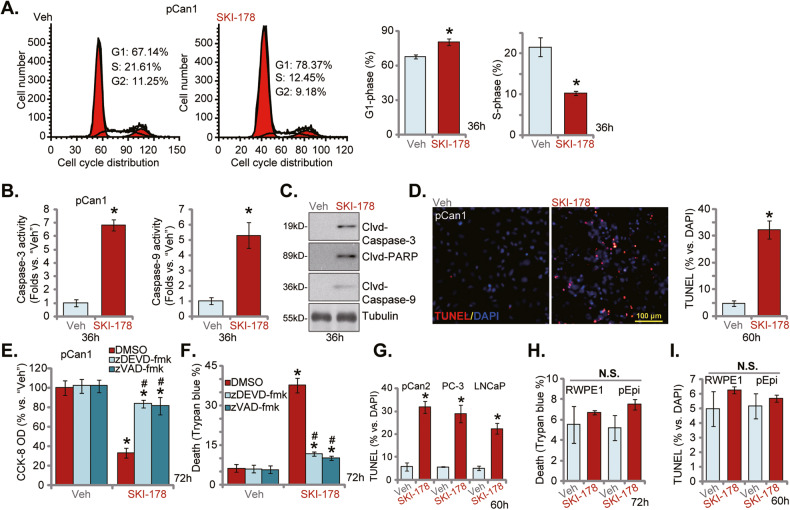
SKI-178 induces cell cycle arrest and apoptosis in prostate cancer cells. The primary human prostate cancer cells (“pCan1” and “pCan2”) (**A**–**D**, **G**), the established cell lines (PC-3 and LNCaP, **G**), the primary human prostate epithelial cells (“pEpi”) (**H**, **I**) or the established RWPE-1 epithelial cells (**H** and **I**) were cultivated in complete medium and treated with SKI-178 (10 μM) for indicated time periods; cell cycle progression (PI-FACS assays, **A**) and caspase-PARP activation (**B**, **C**) were tested; Cell apoptosis was examined by the nuclear TUNEL staining (**D**, **G** and **I**) assays, with cell death measured by Trypan blue staining (**H**). The pCan1 primary cells were pretreated for 1 h with z-DEVD-fmk (40 μM) or z-VAD-fmk (40 μM), followed by SKI-178 (10 μM) stimulation for 72 h; Cell viability and death were tested by CCK-8 (**E**) and Trypan blue staining (**F**) assays, respectively. “Veh” stands for vehicle control. Values represented the mean ± SD (*n* = 5). *P < 0.05 versus “Veh” group. ^#^*P* < 0.05 versus SKI-178 only treatment (**E**, **F**). Experiments in this figure were repeated five times, with similar results achieved. Scale Bar = 100 μm.

The PI-FACS assay was employed to examine cell cycle distribution. In SKI-178 (10 μM, 36 h)-treated pCan1 primary cells, the G1 phase cell percentage was significantly increased (Fig. 3A) and the S-phase cell percentage was robustly decreased (Fig. 3A). These results implied that the SphK1/2 dual inhibitor possibly induced G1-S arrest. SphK1/2 inhibition could induce apoptosis activation in prostate cancer cells [11, 26–29]. As shown the caspase-3 activity and the caspase-9 activity were both significantly increased in SKI-178 (10 μM)-treated pCan1 cells (Fig. 3B). SKI-178 also induced cleavages of caspase-3, caspase-9 and poly(ADP-ribose) polymerase (PARP) in pCan-1 primary cells (Fig. 3C). To support apoptosis activation, we found that the TUNEL-positive nuclei ratio was significantly increased in SKI-178-treated pCan1 primary cells (Fig. 3D). Importantly, the caspase-3 inhibitor z-DEVD-fmk [30] and the pan caspase inhibitor z-VAD-fmk [31] largely ameliorated SKI-178 (10 μM)-induced viability (CCK-8 OD) reduction (Fig. 3E) and cell death (Fig. 3F).

Whether apoptosis was induced by SKI-178 in other prostate cancer cells was tested next. As shown, in the pCan-2 primary prostate cancer cells and established lines (PC-3 and LNCaP), the SphK1/2 dual inhibitor induced significant apoptosis activation, evidenced by significantly increased TUNEL-positive nuclei ratio (Fig. 3G). Interestingly, the Trypan blue staining assay results, Fig. 3H, showed that SKI-178 did not induce significant cell death in RWPE-1 cells and primary epithelial cells. The dual inhibitor also failed to significantly increase the TUNEL-positive nuclei ratio (Fig. 3I).

### The mitochondrial functions are impaired following SKI-178 treatment in prostate cancer cells

**Fig. 4:**
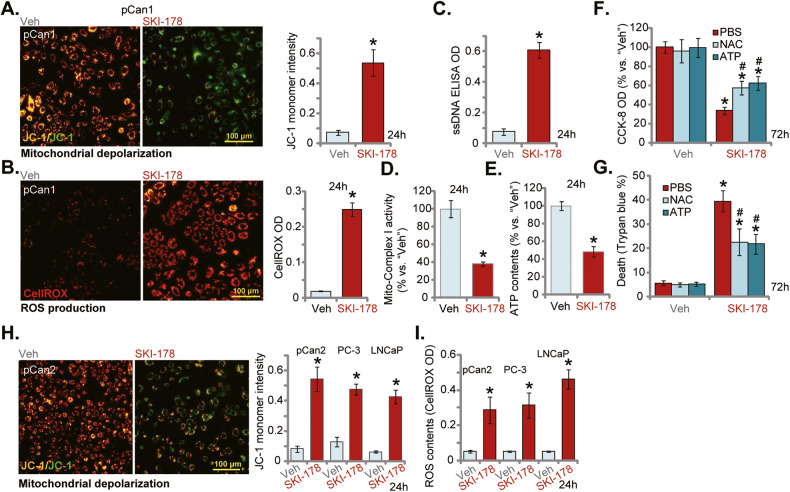
The mitochondrial functions are impaired following SKI-178 treatment in prostate cancer cells. The primary human prostate cancer cells (“pCan1”) cells were cultivated in complete medium and treated with SKI-178 (10 μM) for 24 h, mitochondrial depolarization (by analyzing JC-1 green monomers, **A**), ROS contents (by examining CellROX fluorescence intensity, **B**), and single strand DNA (ssDNA) contents (ELISA OD, **C**) as well as the mitochondrial complex I activity (**D**) and the cellular ATP contents (**E**) were tested. The pCan1 primary cells were pretreated for 1 h with NAC (500 μM) or ATP (2 mM), followed by SKI-178 (10 μM) stimulation for72h; Cell viability and death were tested by CCK-8 (**F**) and Trypan blue staining (**G**) assays, respectively. The primary human prostate cancer cells (“pCan2”) or established cell lines (PC-3 and LNCaP) were cultivated in complete medium and treated with SKI-178 (10 μM) for 48 h; Mitochondrial depolarization (by analyzing JC-1 green monomers, **H**) and ROS contents (by examining CellROX fluorescence intensity, **I**) were tested. “Veh” stands for vehicle control. Values represented the mean ± SD (*n* = 5). *P < 0.05 versus “Veh” group. ^#^*P* < 0.05 versus SKI-178 only treatment (**F**, **G**). Experiments in this figure were repeated five times, with similar results achieved. Scale Bar = 100 μm.

Results in Fig. 3 demonstrated that treatment with SKI-178 resulted in significant apoptosis activation, leading to prostate cancer cell death. Moreover, supporting mitochondrial-dependent apoptosis cascade activation, robust caspase-3 and caspase-9 activation in SKI-178-treated prostate cancer cells was detected (Fig. 3). Next, we explored whether mitochondrial functions were impaired by the SphK1/2 dual inhibitor. JC-1 assay results in Fig. 4A showed that SKI-178 stimulation (10 μM) led to JC-1 green monomer accumulation in pCan1 primary cells, indicating mitochondrial depolarization [32]. Significantly, SKI-178 treatment caused ROS production and oxidative injury in pCan1 cells. The CellROX red fluorescence intensity (Fig. 4B) was substantially increased in pCan1 cells after SKI-178 treatment. Moreover, the dual inhibitor induced robust DNA breaks and increased ssDNA contents (Fig. 4C). Further supporting impairment of mitochondrial functions, we found that the activity of mitochondrial complex I was decreased in SKI-178-stimulated pCan1 cells (Fig. 4D). Consequently, the cellular ATP contents were reduced (Fig. 4E). These results supported that SKI-178 impaired mitochondrial functions in pCan1 primary cells, causing mitochondrial depolarization, ROS production, oxidative injury and ATP reduction.

Whether mitochondrial disruption played an important role in SKI-178-induced prostate cancer cell death was studied next. As shown the well-known anti-oxidant NAC and supplementing ATP attenuated SKI-178 (10 μM)-induced CCK-8 viability reduction (Fig. 4F) and cell death (Fig. 4G) in pCan1 cells. These results supported that mitochondrial disruption should be one important contributor of SKI-178-induced prostate cancer cell death. In pCan2 primary prostate cancer cells and established lines (PC-3 and LNCaP), treatment with SKI-178 (10 μM) also induced mitochondrial depolarization (JC-1 green monomer accumulation, Fig. 4H) and ROS accumulation (CellROX intensity increasing, Fig. 4I), further supporting impairment of mitochondrial functions.

### SKI-178 inhibits SphK1 and SphK2 activity in prostate cancer cells

**Fig. 5:**
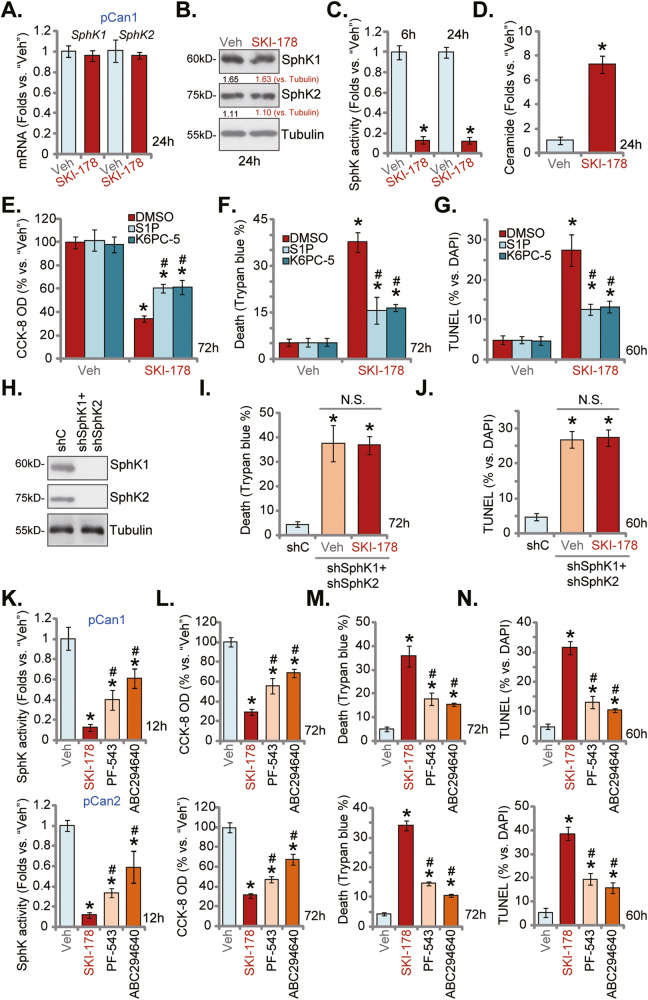
SKI-178 inhibits SphK1 and SphK2 activity in prostate cancer cells. The primary human prostate cancer cells (“pCan1”) were treated with SKI-178 (10 μM) for applied time periods, mRNA and protein expression of SphK1 and SphK2 were tested by qRT-PCR (**A**) and Western blotting (**B**) assays, and SphK activity tested using the described methods (**C**). Cellular ceramide contents were examined as well (**D**). The pCan1 primary cells were pretreated for 1 h with S1P (25 μM), K6PC-5 (10 μM) or the vehicle control (“DMSO”), followed by SKI-178 (10 μM) stimulation for applied time periods, cell viability, death and apoptosis were tested by CCK-8 (**E**), Trypan blue staining (**F**) and nuclear TUNEL staining (**G**) assays, respectively. The pCan1 primary cells bearing the lentiviral SphK1 shRNA and the lentiviral SphK2 shRNA (“shSphK1+shSphK2”) were treated with SKI-178 (10 μM) or vehicle, control cells were transduced with the scramble control shRNA (“shC”), expression of listed proteins were shown (**H**). Cells were further cultured for applied time periods, cell death and apoptosis were tested by Trypan blue staining (**I**) and nuclear TUNEL staining (**J**) assays, respectively. The primary human prostate cancer cells (“pCan1” and “pCan2”) were treated with 10 μM of SKI-178, PF-543 or ABC294640 and cultured for applied time periods, the SphK1 activity was shown (**K**); Cell viability, cell death and apoptosis were tested by CCK-8 (**L**), Trypan blue staining (**M**) and TUNEL staining (**N**) assays, respectively. “Veh” stands for vehicle control. Values represented the mean ± SD (*n* = 5). *P < 0.05 versus “Veh”/“shC” group. ^#^*P* < 0.05 versus SKI-178 group. “N.S.” stands for non-statistical difference. Experiments in this figure were repeated five times, with similar results achieved.

Since SKI-178 is a novel and highly efficient SphK1/2 dual inhibitor [12, 14], we next analyzed SphK1/2 expression and activity in SKI-178-treated prostate cancer cells. As shown treatment with SKI-178 (10 μM, 24 h) failed to affect mRNA expression of *SphK1* and *SphK2* in pCan1 primary cancer cells (Fig. 5A). SphK1 and SphK2 protein expression, tested by Western blotting assays, was not significantly altered as well (Fig. 5B). Conversely, the SphK activity was significantly decreased in SKI-178-treated pCan1 primary cells (Fig. 5C). SphK activity reduction started at 6 h after SKI-178 treatment and lasted for at least 24 h (Fig. 5C). Consequently, the cellular ceramide levels were significantly increased in pCan1 cells with SKI-178 treatment (Fig. 5D).

Experiments were then performed to test whether SphK inhibition is the main reason of SKI-178-induced cytotoxicity in prostate cancer cells. K6PC-5, a direct SphK1 activator [33–36] and sphingosine-1-phosphate (S1P) were added. As shown, in pCan1 cells pretreatment with K6PC-5 or S1P potently inhibited SKI-178-induced viability (CCK-8 OD) reduction (Fig. 5E), cell death (Trypan blue staining increase, Fig. 5F) and apoptosis (TUNEL-positive nuclei ratio increase, Fig. 5G).

Next, the lentiviral SphK1 shRNA and the lentiviral SphK2 shRNA were co-added to pCan1 cells. Via selection by puromycin stable cells were established, namely “shSphK1+shSphK2” cells. SphK1 and SphK2 protein levels were silenced in the “shSphK1+shSphK2” pCan1 cells (Fig. 5H). Mimicking SKI-178-induced actions, shRNA-induced silencing of SphK1 and SphK2 led to dramatic cell death and apoptosis, tested by Trypan blue staining (Fig. 5I) and nuclear TUNEL staining (Fig. 5J) assays, respectively. Significantly, treatment with SKI-178 (10 μM, 72 h) failed to induce further cytotoxicity (Fig. 5I) and apoptosis (Fig. 5J) in “shSphK1+shSphK2” pCan1 cells. Therefore, SKI-178 was invalid in SphK1/2-silenced cells, supporting that SphK inhibition should be the primary mechanism of SKI-178-induced cytotoxicity in prostate cancer cells.

We also compared the anti-prostate cancer cell activity between SKI-178 and other SphK1/2 inhibitors, including the SphK1 inhibitor PF-543 [37] and the SphK2 inhibitor ABC294640 [38–41]. In pCan1 and pCan2 primary prostate cancer cells SKI-178-induced SphK inhibition (Fig. 5K), viability (CCK-8 OD) reduction (Fig. 5L), cell death (Trypan blue staining assays, Fig. 5M) and apoptosis (TUNEL-positive nuclei ratio increase, Fig. 5N) were significantly more potent than the same concentration of PF-543 and ABC294640. These results implied that co-current inhibition of SphK1 and SphK2 by SKI-178 should led to more significant anti-prostate cancer cell activity then single blockage.

### SKI-178 inhibits Akt-mTOR activation in prostate cancer cells

**Fig. 6:**
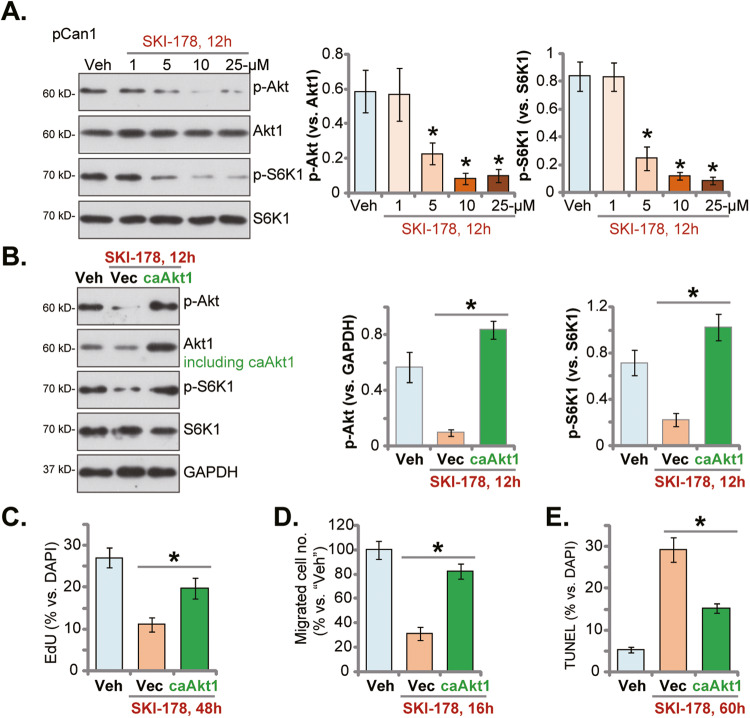
SKI-178 inhibits Akt-mTOR activation in prostate cancer cells. The primary human prostate cancer cells (“pCan1”) cells were cultivated in complete medium and treated with SKI-178 (at designated concentrations) for 12 h, expression of listed proteins was shown (**A**). pCan1 cells, expressing the lentivirus-packed constitutively-active mutant Akt (S473D, “caAkt1”) or the empty vector (“Vec”), were treated with SKI-178 (10 μM) for 12 h, expression of listed proteins was shown (**B**). Alternatively, cells were cultivated for indicated time periods, cell proliferation (by measuring nuclear EdU ratio, **C**) and cell migration (“Transwell” assays, **D**) were tested. Cell apoptosis was examined via measuring nuclear TUNEL ratio (**E**) assays. Values represented the mean ± SD (*n* = 5). *P < 0.05 versus “Veh” group (**A**). *P < 0.05 (**B**–**E**). Experiments in this figure were repeated five times, with similar results achieved.

It is known that SphK1 inhibition causes ceramide production to inhibit Akt phosphorylation by activating phosphotases, including protein phosphatase 1 (PP1) and protein phosphatase 2 A (PP2A) [42–44]. Considering that activation of Akt is vital for the progression of prostate cancer [16, 45–47], we examined whether SKI-178 could inhibit activation of Akt and its major downstream mTOR in prostate cancer cells. As shown, treatment with SKI-178 dose-dependently inhibited phosphorylation of Akt and S6K1 in pCan1 cells (Fig. 6A). Akt and S6K1 phosphorylation inhibition was significantly after 5-25 μM of SKI-178 treatment (Fig. 6A). Expression of total Akt and S6K1 was unchanged in SKI-178-treated pCan1 cells (Fig. 6A). Next the contribution of Akt-mTOR inhibition on SKI-178-induced anti-prostate cancer cell activity was studied. Specifically, the lentivirus-packed constitutively-active mutant Akt (S473D), caAkt1 [18, 44, 48], was stably transduced to pCan1 primary cancer cells, which completely restored Akt and S6K1 phosphorylation in SKI-178 (10 μM)-treated cells (Fig. 6B). Functional studies revealed that caAkt1 alleviated SKI-178 (10 μM)-induced proliferation inhibition (EdU ratio decline, Fig. 6C) and migration reduction (Fig. 6D) in pCan1 cells. Moreover, SKI-178 (10 μM)-induced pCan1 cell apoptosis (TUNEL-nuclei ratio increasing) was ameliorated by caAkt1 (Fig. 6E). These results supported that Akt-mTOR inactivation should be one important mechanism of SKI-178-induced anti-prostate cancer cell activity.

### SKI-178 activates JNK cascade in prostate cancer cells

**Fig. 7:**
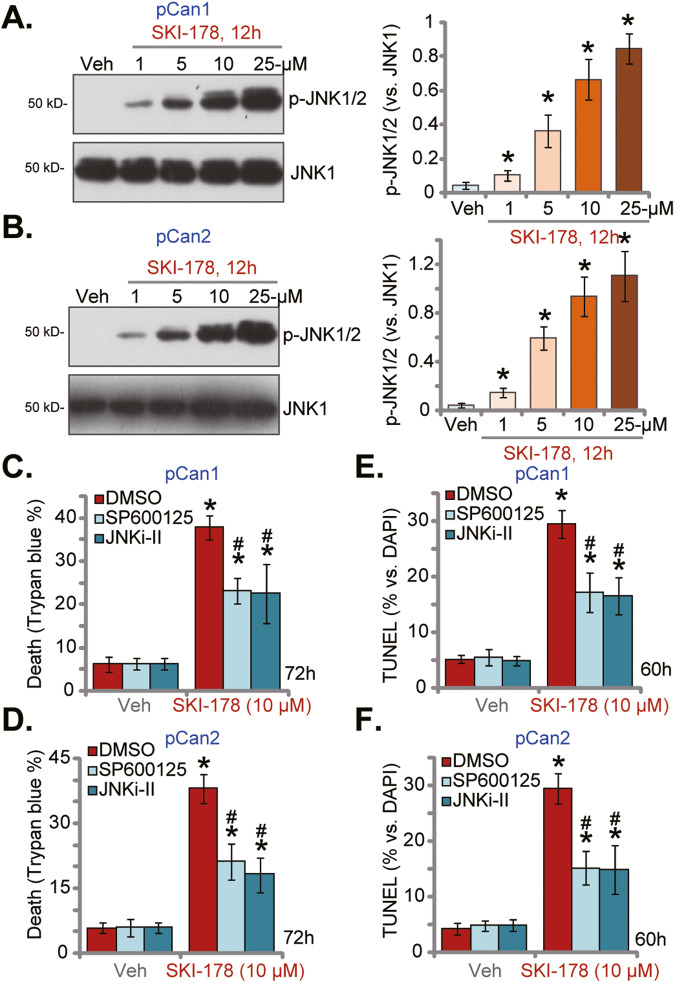
SKI-178 activates JNK cascade in prostate cancer cells. The primary human prostate cancer cells (“pCan1/pCan2”) cells were cultivated in complete medium and treated with SKI-178 (at designated concentrations) for 12 h, expression of listed proteins was shown (**A**, **B**). The pCan1 and pCan2 primary cells were pretreated for 1 h with SP600125 (10 μM) or JNKi-II (10 μM), followed by SKI-178 (10 μM) stimulation for 60 h/72 h; Cell death and apoptosis were Trypan blue staining (**C**, **D**) and TUNEL nuclei staining (**E**, **F**) assays, respectively. Values represented the mean ± SD (*n* = 5). *P < 0.05 versus “Veh” group. ^#^*P* < 0.05 versus SKI-178 only treatment (**C**–**F**). Experiments in this figure were repeated five times, with similar results achieved.

Ceramide accumulation after SphK inactivation could also provoke JNK activation, thereby promoting cancer cell apoptosis and death [13, 39, 43, 49, 50]. We therefore analyzed whether SKI-178 could activate JNK cascade in prostate cancer cells. As shown JNK activation was significantly increased after SKI-178 treatment in pCan1 and pCan2 primary cancer cells (Fig. 7A and B). SKI-178 dose-dependently increased JNK1/2 phosphorylation in pCan1 and pCan2 cells (Fig. 7A, B). To block JNK activation, two well-established JNK inhibitors were utilized, including SP600125 and JNKi-II. As shown, SKI-178 (10 μM)-induced cell death, or Trypan blue positive staining, was inhibited following co-treatment with JNK inhibitors in pCan1 (Fig. 7C) and pCan2 (Fig. 7D) cells. Moreover, the nuclear TUNEL staining assay results confirmed that JNK inhibition alleviated apoptosis activation in SKI-178 (10 μM)-treated pCan1 cells (Fig. 7E) and pCan2 cells (Fig. 7F). These results supported JNK activation participated in SKI-178-induced apoptosis in prostate cancer cells.

### SKI-178 administration inhibits PC-3 xenograft growth in nude mice

**Fig. 8:**
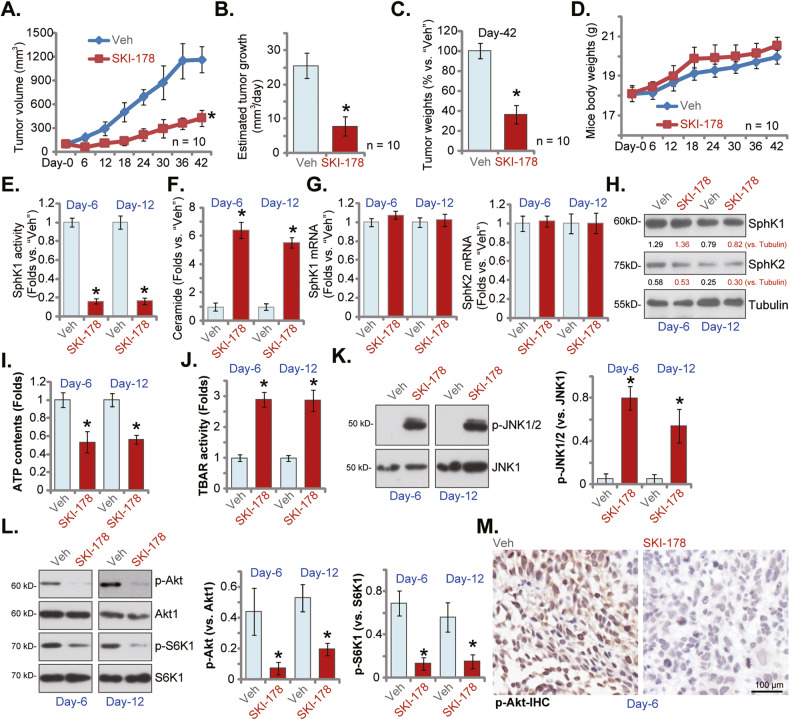
SKI-178 administration inhibits PC-3 xenograft growth in nude mice. PC-3 xenograft-bearing nude mice were administrated with SKI-178 (intraperitoneal injection, 25 mg/kg body weight, daily for 18 days) or vehicle control (“Veh”); Tumor volumes (**A**) and mice body weights (**D**) were recorded every 6 days for a total of 42 days (“Day-0” to “Day-42”). Estimated daily tumor growth, in mm^3^ per day, was calculated as described (**B**). At “Day-42” all tumors were isolated and weighted (**C**). At Day-6 and Day-12, one tumor of each group was isolated carefully, and tumor tissues from the total four tumors were obtained; Each tumor was cut into five pieces, SphK activity (**E**), ceramide contents (**F**) and expression of listed mRNAs and proteins (**G**, **H**, **K** and **L**) as well as ATP contents (**I**) and TBAR activity (**J**) were tested. Alternatively, p-Akt (Ser-473) in the PC3 xenograft slides was tested by immunohistochemistry (IHC) staining (**M**). “Veh” stands for vehicle control. Values represented the mean ± SD. *P < 0.05 versus “Veh” group.

At last we tested the potential anti-cancer activity of SKI-178 in vivo. A significant number of PC-3 cells (6 × 10^6^ cells per mouse) were subcutaneously (*s.c*.) injected to the flanks of the nude mice. Within 20 days, the PC-3 xenografts were established (labeled as “Day-0”) with tumro volumes close to 100 mm^3^. The PC-3 xenograft-bearing mice were thereafter randomly assigned into two groups, with 10 mice per group (*n* = 10). The SKI-178 treatment mice received intraperitoneal injection of SKI-178 (25 mg/kg body weight), daily for 18 days. The control group mice received vehicle control administration (“Veh”). In Fig. 8A the tumor growth curve results showed that SKI-178 administration potently inhibited PC-3 xenograft growth in nude mice. The volumes of PC-3 xenografts with SKI-178 treatment were significantly lower than those with vehicle administration (Fig. 8A). The estimated daily tumor growth was calculated by the following formula: (Tumor volume at “Day-42” subtracting tumor volume at “Day-0”)/42. Results again showed that SKI-178 administration robustly inhibited PC-3 xenograft growth in nude mice (Fig. 8B). At Day-42 PC-3 xenografts of the two groups were isolated carefully and weighted. We showed that SKI-178-treated PC-3 xenografts were significantly lighter than vehicle-treated xenografts (Fig. 8C). Results in Fig. 8D showed that SKI-178 administration failed to significantly alter body weights of the experimental mice. These results clearly showed that daily intraperitoneal injection of SKI-178 potently inhibited PC-3 xenograft tumor growth in nude mice, demonstrating its anti-cancer activity in vivo.

To analyze signaling changes in vivo, at Day-6 and Day-12, one tumor of each group was isolated carefully. The tumor tissues from the four tumors were examined. In Fig. 8E, we showed that the SphK activity decreased over 80% in SKI-178-treated PC-3 xenograft tissues. In contrast, ceramide contents were significantly increased in PC-3 xenograft tissues with SKI-178 administration (Fig. 8F). The qRT-PCR assay results, Fig. 8G, found that mRNA expression of *SphK1* and *SphK2* in PC-3 xenograft tissues was unchanged after SKI-178 injection. Moreover, SphK1 and SphK2 protein expression was not altered as well (Fig. 8H). Therefore, in line with the in vitro findings, SKI-178 administration inhibited SphK activity without affecting SphK1 /2 expression in PC-3 xenografts.

Importantly, the ATP contents were decreased in SKI-178-administrated PC-3 xenograft tissues (Fig. 8I). Supporting lipid lipid peroxidation the TBAR activity was substantially increased in PC-3 xenograft tissues following SKI-178 treatment (Fig. 8J). These results supported that SKI-178 administration impaired mitochondrial functions in PC-3 xenograft tissues. In addition, SKI-178 activated JNK cascade in vivo and phosphorylated JNK1/2 levels were significantly increased in SKI-178-treated PC-3 xenograft tissues (Fig. 8K). Contrarily, phosphorylation of Akt and S6K1 was largely decreased in PC-3 xenograft tissues after SKI-178 administration (Fig. 8L). The immunohistochemistry (IHC) staining assay in PC-3 xenograft slides results further supported that SKI-178 inhibited Akt activation in vivo (Fig. 8M). Thus, in line with the signaling results in vitro, SKI-178 administration induced mitochondrial dysfunction, JNK activation and Akt-mTOR inhibition in PC-3 xenografts.

### SphK1 and SphK2 overexpression promotes primary human prostate cancer cell growth in vivo

**Fig. 9:**
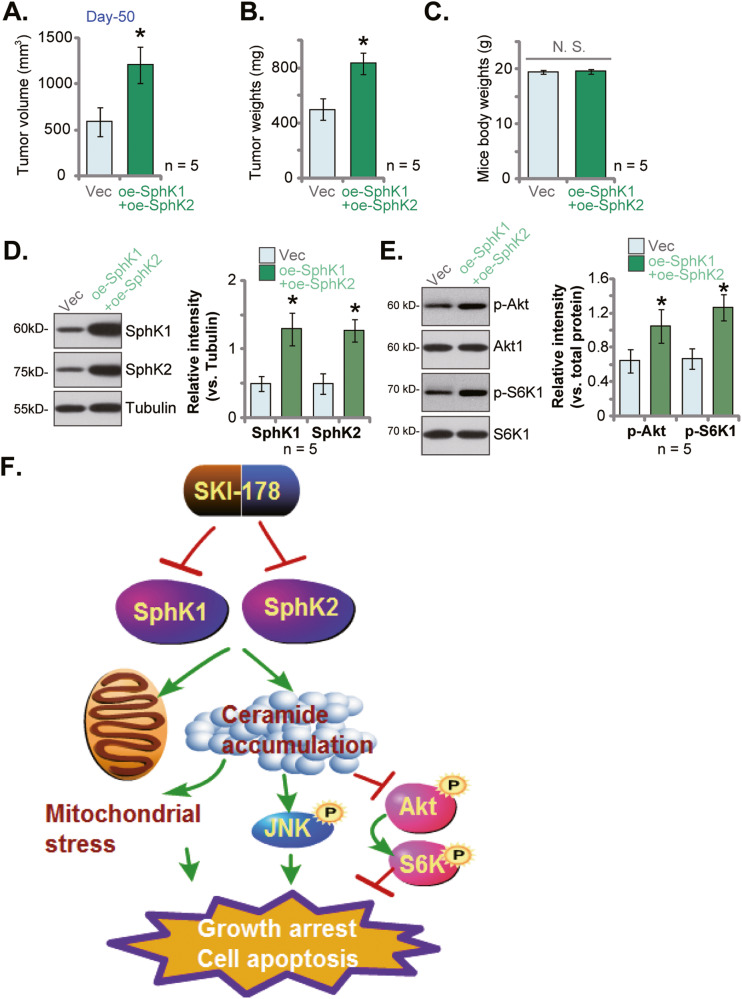
SphK1 and SphK2 overexpression promotes primary human prostate cancer cell growth in vivo. The primary human prostate cancer cells pCan1, with the lentiviral SphK1-overexpressing construct plus the lentiviral SphK2-overexpressing construct (“oe-SphK1+oe-SphK2”) or the empty vector (“Vec”), were *s.c*. injected to the flanks of the nude mice (at six million cells per mouse). After 50 days, the tumor volumes (**A**), the tumor weights (**B**) and the mice body weights (**C**) were recorded. Expression of listed proteins in tumor tissues were tested (**D**, **E**). The proposed signaling cartoon of this study (**F**). Values represented the mean ± SD (five xenografts per group, *n* = 5). *P < 0.05 versus “Vec” group. “N.S.” stands for non-statistical difference.

To further support the role of SphK1/2 in prostate cancer cell growth in vivo, the oe-SphK1+oe-SphK2 pCan1 cells (see Fig. 1) or the vector control pCan1 cells (“Vec”, also see Fig. 1) were *s.c*. injected the nude mice. Fifty (50) days after cell injection, xenografts were measured. The volumes of oe-SphK1+oe-SphK2 pCan1 xenografts were significantly higher than those of vector control pCan1 xenografts (Fig. 9A). Moreover, the oe-SphK1+oe-SphK2 pCan1 xenografts were heavier than the vector control xenografts (Fig. 9B). The mice body weights were not significantly different between the two groups (Fig. 9C). When analyzing the pCan1 xenograft tissues, we showed that SphK1 and SphK2 protein levels were elevated in oe-SphK1+oe-SphK2 pCan1 xenografts (Fig. 9D) and Akt-S6K1 phosphorylation was increased (Fig. 9E). These results showed that ectopic overexpression of SphK1 and SphK2 further promoted primary human prostate cancer cell growth in vivo.

## Discussion

Prostate cancer is the fifth leading cancer-caused death among men [1, 2]. It is extremely important to further understand the pathological mechanism for prostate cancer progression [3, 51–53]. The clinical trials testing inhibitors of PI3K and ERK pathways against prostate cancer have failed [51, 52]. The pan-RTK (receptor tyrosine kinase) inhibitor cabozantinib and VEGFR inhibitors (aflibercept and bevacizumab) have entered stage III clinical studies trials, yet their clinical benefits are not satisfactory [3, 51–53].

Recent studies have shown that SphK inhibition by the pharmacological agents could potently inhibit prostate cancer cell growth. Pchejetski et al., have shown that FTY720 inhibited SphK1 to sensitize prostate cancer cells to radiotherapy [26]. FTY720 single treatment also induced prostate cancer cell apoptosis by inhibiting SphK1 and inducing ceramide production [26]. Dayon et al., showed that chronic androgen deprivation induced SphK1 activation, acting as a compensatory mechanism to promote prostate cancer cell survive [54]. Conversely, SphK1 inhibition significantly augmented androgen deprivation-induced anti-prostate cancer cell activity [54]. Moreover, SphK2 inhibition by ABC294640 inhibited viability and proliferation in androgen resistant prostate cancer cells [11].

SKI-178 is a small-molecule, non-lipid and highly-efficient SphK1/2 dual inhibitor [15]. It has displayed significant activities in different human cancer cells [12–14]. Hengst et al., showed that SKI-178 simantanuously blocked SphK1 and SphK2 and disrupted microtubule dynamics, inhibiting acute myeloid leukemia cell growth in vitro and in vivo [12]. Dick et al., found that SKI-178 not only inhibited SphK activity but also induced sustained activation of cyclin-dependent protein kinase 1 (CDK1), causing apoptosis and growth arrest in AML cells [14]. LeBlanc et al., found that SphK inhibition by SKI-178 led to ceramide production, phosphorylations CDK1 and Bcl-2, as well as decreased JAK-STAT signaling, and eventually causing cell cycle arrest and apoptosis in natural killer-large granular lymphocyte leukemia cells [13].

Here the TCGA database results and results from local tissues demonstrated that that both SphK1 and SphK2 are upregulated in human prostate cancer tissues. Targeting SphK1/2 by SKI-178 induced significant anti-prostate cancer activity. In primary human prostate cancer cells and established cell lines, SKI-178 potently inhibited cell viability, proliferation, cell cycle progression and cell migration. Significant cell death and apoptosis were detected in SKI-178-treated primary and established prostate cancer cells. It however failed to induce significant cytotoxicity in prostate epithelial cells. In vivo, daily intraperitoneal injection of a single dose of SKI-178 potently inhibited PC-3 xenograft growth in nude mice. Contrarily, ectopic overexpression of SphK1 and SphK2, by lentiviral constructs, further increased promoted primary prostate cancer cell growth in vitro and in vivo.

We here provided evidence that SphK inhibition should be the primary cause of SKI-178-induced activity in prostate cancer cells. SKI-178 largely inhibited SphK activity and induced ceramide production, without affecting SphK1/2 expression, in prostate cancer cells. K6PC-5, the SphK1 activator, or supplement with S1P, largely ameliorated SKI-178-induced cytotoxicity in primary prostate cancer cells. Significantly, shRNA-induced silencing of SphK1/2 not only mimicked SKI-178-induced actions in prostate cancer cells. More importantly, SKI-178 was unable to induce further prostate cancer cell apoptosis. Since SKI-178 can block both SphK1 and SphK2, it was not surprising to show that SKI-178-induced SphK inhibition and prostate cancer cell death were significantly more potent than the SphK1 inhibitor PF-543 and the SphK2 inhibitor ABC294640. The SphK1/2 dual inhibitor also failed to induce significant cytotoxicity in SphK1/2-low prostate epithelial cells. Further, SKI-178 failed to induce apparent toxicity to the nude mice. These results suggested that SKI-178 could be a promising therapeutic target for prostate cancer.

Studies have shown that SphK1 inactivation/silencing could result in ceramide accumulation in cancer cells, which then inactivates Akt-mTOR cascade by activating phosphatases PP2A and PP1 [39, 43, 55, 56]. Xue et al., found that SKI-349, a novel, highly efficient and small molecular SphK1/2 dual inhibitor, inhibited Akt-mTOR activation in non-small cell lung cancer cells [43]. Sun et al., revealed that SKI-V, a non-lipid SphK1 inhibitor, blocked Akt-mTOR activation in primary human osteosarcoma cells [44]. ABC294640, a SphK2 specific inhibitor, also induced ceramide production and Akt-mTOR inactivation, thereby inhibiting colorectal cancer cell growth in vitro and in vivo [39]. In hepatocellular carcinoma cells, SphK1 inhibition by cinobufotalin induced ceramide production and Akt-mTOR inhibition [55]. In the present study we found that SphK1/2 dual inhibition by SKI-178 caused Akt-mTOR inactivation in prostate cancer cells. Akt-mTOR inactivation was observed in SKI-178-treated PC-3 xenograft tissues as well. Importantly, SKI-178-induced anti-prostate cancer cell activity, including proliferation inhibition, migration slowing and apoptosis activation, were alleviated by caAkt1. The latter restored Akt-mTOR activation in SKI-178-treated prostate cancer cells. Interestingly, ectopic overexpression of SphK1 and SphK2 further increased Akt-mTOR activation in primary prostate cancer cells. These results supported that Akt-mTOR inactivation is one important mechanism of SKI-178’s actions in prostate cancer cells (Fig. 9F).

Sustained activation of JNK cascade could cause growth inhibition and apoptosis in prostate cancer cells [57]. Shi et al., reported that TNFα provoked JNK cascade to promote prostate cancer cell apoptosis [58]. Lv et al., reported that silence of serine/threonine protein phosphatase 5 (PPP5C) inhibited proliferation of prostate cancer cells via activating JNK cascade [59]. Xie et al., found that prostate-specific G protein-coupled receptor (PSGR) activation by β-ionone provoked JNK signaling pathway and inhibited prostate cancer cell growth [60]. SphK inhibition and ceramide accumulation could also initiate JNK activation [61–63]. Here we found that SKI-178 activated JNK cascade in primary human prostate cancer cells. Moreover, JNK activation was also detected in SKI-178-administrated PC-3 xenograft tissues. JNK inhibition by pharmacological inhibitors attenuated SKI-178-induced prostate cancer cell death and apoptosis. These results support that JNK activation is an important contributor of SKI-178-induced anti-cancer actions in prostate cancer cells (Fig. 9F).

## Conclusion

SphK1/2 inhibition by SKI-178 suppresses prostate cancer cell growth in vitro and in vivo.

## Supplementary information

Original Data File

aj-checklist FORM

Author contribution FORM

## Data Availability

All data are included in the article. Further inquiries can be directed to the corresponding authors.
