# Supplementary figures and images for: Targeting SphK1/2 by SKI-178 inhibits prostate cancer cell growth
Source: Cell Death Dis. 2023 Aug 21;14(8):537. doi: 10.1038/s41419-023-06023-4 (PMC10442381; doi:10.1038/s41419-023-06023-4)

Figure S1. The uncropped blotting images

Figure 1

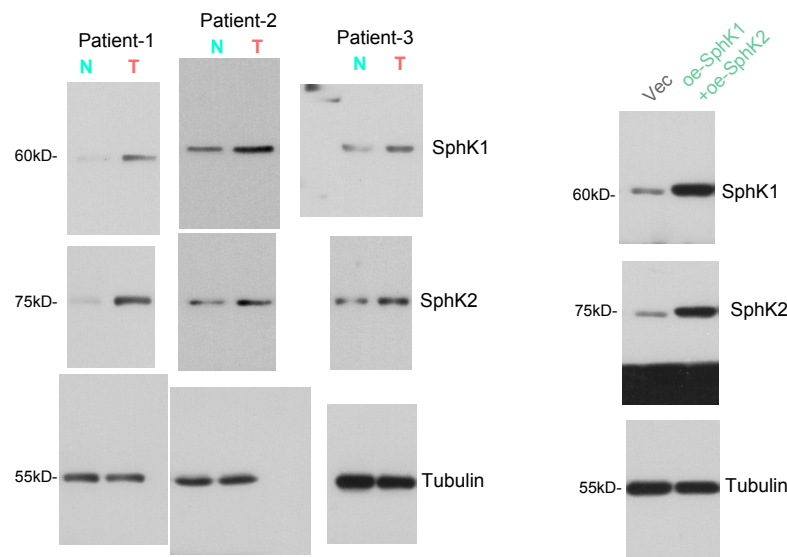

Figure 3

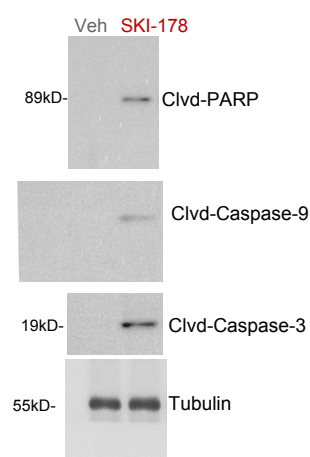

Figure 5

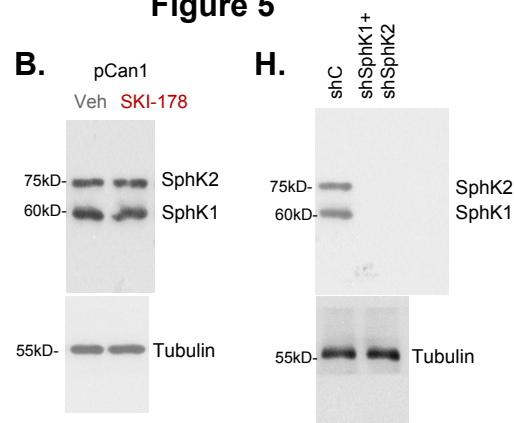

Figure 7

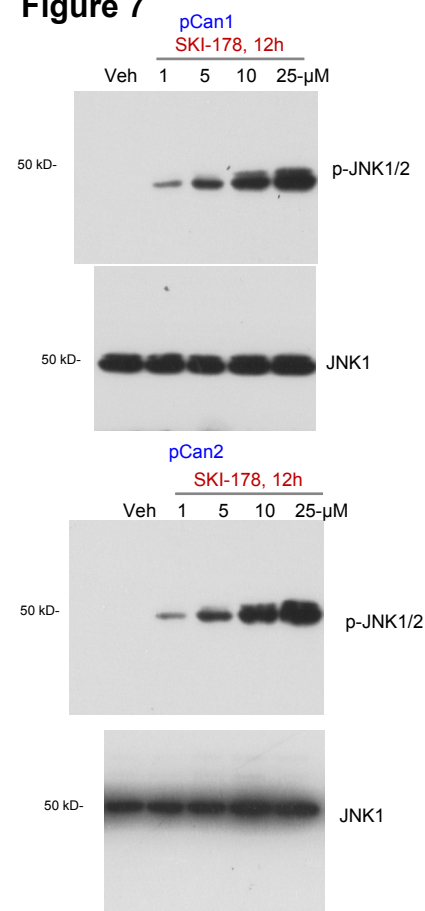

Figure 6.

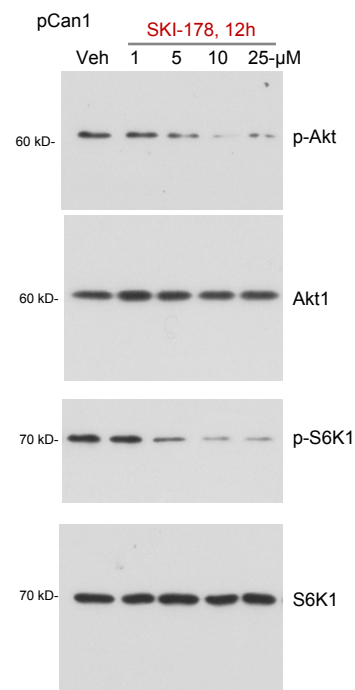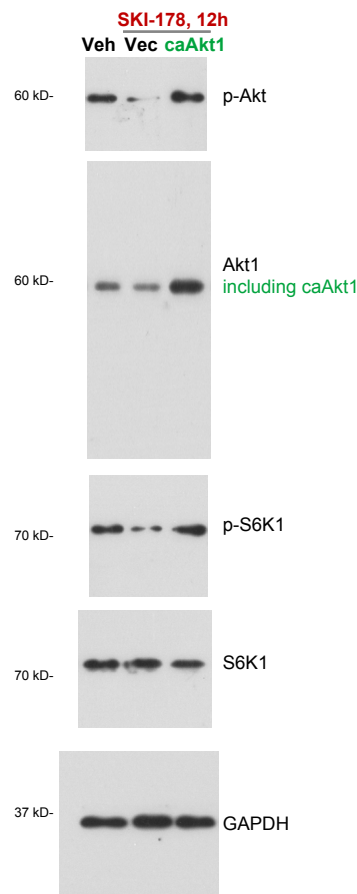

**Figure 8**

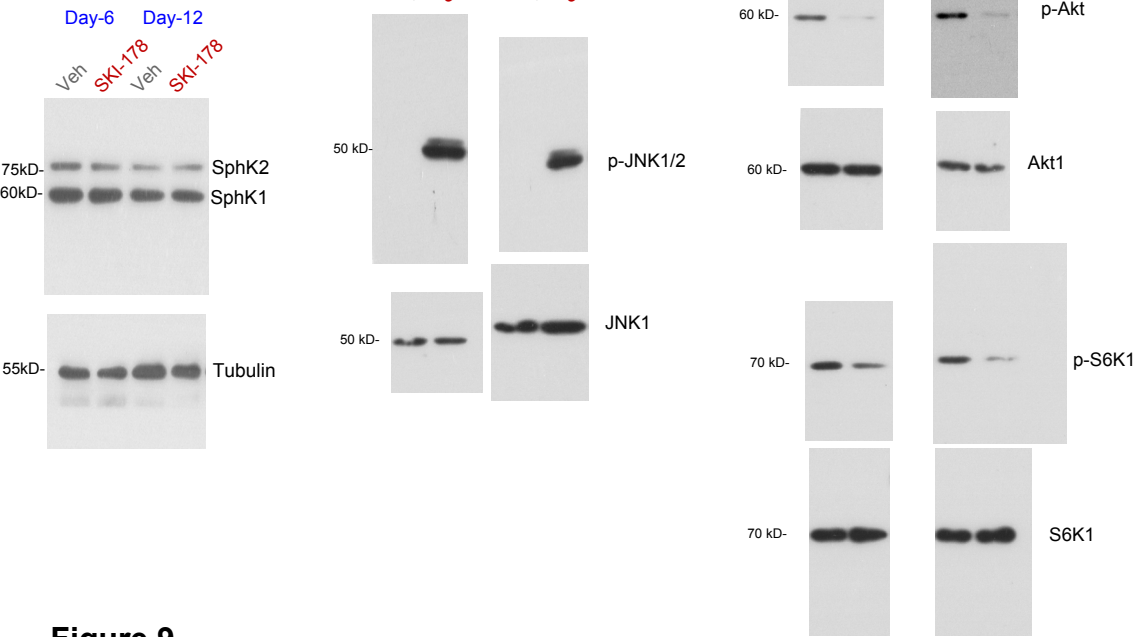

**Figure 9**

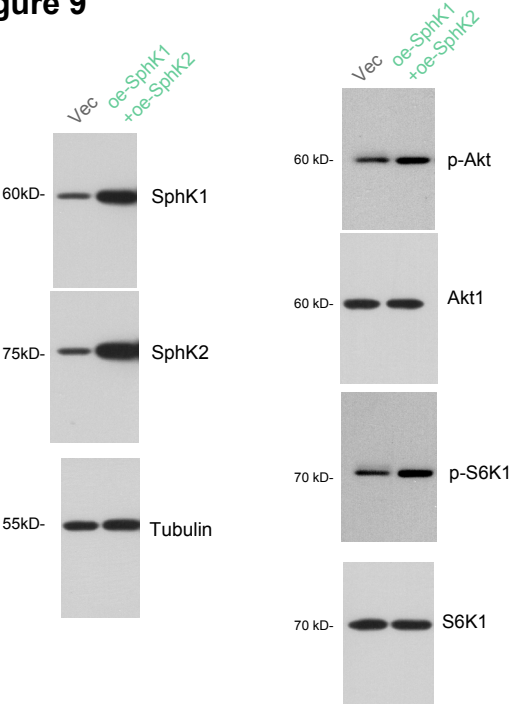

Supplement: Supplementary file 1 — Original Data File [file 41419_2023_6023_MOESM1_ESM.pdf]
