# Supplementary material for: Targeting SphK1/2 by SKI-178 inhibits prostate cancer cell growth
Source: Cell Death Dis. 2023 Aug 21;14(8):537. doi: 10.1038/s41419-023-06023-4 (PMC10442381; doi:10.1038/s41419-023-06023-4)
Supplement: Supplementary file 3 — Author contribution FORM [file 41419_2023_6023_MOESM3_ESM.pdf]

DECLARATION OF CONTRIBUTIONS TO ARTICLE

ADMC

Manuscript Number: CDDIS-23-1517R

Journal Name: *Cell Death & Disease* (the 'Journal')

Proposed Title of the Contribution:

Targeting SphK1/2 by SKI-178 inhibits prostate cancer cell growth (the 'Contribution)

Author(s):

Lu Jin, Jin Zhu, Linya Yao, Gang Shen, Bo-xin Xue and Wei Tao (the 'Authors')

For all *CDDis* articles, each person named as an author in the published version must be able to show he or she has contributed substantially to the article.

Authorship credit should be based on 1) substantial contributions to conception and design, acquisition of data, or analysis and interpretation of data; 2) drafting the article or revising it critically for important intellectual content; and 3) final approval of the version to be published. Authors should meet conditions 1, 2 and 3.

Any person who cannot be shown to have made a substantial contribution to the article cannot be listed as an author in the final version. The name of any person who is deemed to have made a minor contribution can, however, appear in the Acknowledgments section of the article.

Please complete the table below to indicate the contributions of all named authors to the manuscript.

Author Full Name:	Specification of Contribution to the Manuscript:
Lu Jin	Culture primary and established prostate cancer cells and normal cells, testing testing signalings, performed the in vivo studies, drafting the article and revising it, with final approval of the version submitted to the journal. Analyzing human tissues.
Jin Zhu	Culture primary and established prostate cancer cells and normal cells, testing testing signalings, performed the in vivo studies, drafting the article and revising it, with final approval of the version submitted to the journal. Analyzing human tissues.
Linya Yao	Culture primary and established prostate cancer cells and normal cells, testing testing signalings, performed the in vivo studies, drafting the article and revising it, with final approval of the version submitted to the journal. Analyzing human tissues
Gang Shen	Study conception and design, and data analysis, Figure organization, involved in drafting the article and revising it critically for important intellectual content, and with final approval of the version submitted to the journal.
Bo-xin Xue	Study conception and design, and data analysis, Figure organization, involved in drafting the article and revising it critically for important intellectual content, and with final approval of the version submitted to the journal.
Wei Tao	Study conception and design, and data analysis, Figure organization, involved in drafting the article and revising it critically for important intellectual content, and with final approval of the version submitted to the journal.

Please complete the table below to indicate the contributions of all named authors to the figures.

Figure 1:

Lu Jin, Jin Zhu, Linya Yao, Gang Shen, Bo-xin Xue and Wei Tao

Figure 2:

Lu Jin, Jin Zhu, Linya Yao, Gang Shen, Bo-xin Xue and Wei Tao

Figure 3:

Lu Jin, Jin Zhu, Linya Yao, Gang Shen, Bo-xin Xue and Wei Tao

Figure 4:

Lu Jin, Jin Zhu, Linya Yao, Gang Shen, Bo-xin Xue and Wei Tao

Figure 5:

Lu Jin, Jin Zhu, Linya Yao, Gang Shen, Bo-xin Xue and Wei Tao

Figure 6:

Lu Jin, Jin Zhu, Linya Yao, Gang Shen, Bo-xin Xue and Wei Tao

Figure 7-9, Lu Jin, Jin Zhu, Linya Yao, Gang Shen, Bo-xin Xue and Wei Tao

Signed for and on behalf of the Author(s):	Print Name:	Date:
Wei Tao	Wei Tao	06/21/2023
